# Magnitude of response in treatment and control groups within psychedelic trials for psychiatric disorders: A meta-analysis

**DOI:** 10.1192/j.eurpsy.2026.10168

**Published:** 2026-02-18

**Authors:** Shakila Meshkat, Qiaowei Lin, Rachel Sousa-Ho, Ilya Demchenko, Richard J. Zeifman, Howell Fang, Amy C. Reichelt, Yanbo Zhang, Lisa Burback, Olga Winkler, Andrew Greenshaw, Candice M. Monson, Eric Vermetten, Rakesh Jetly, Wendy Lou, Muhammad Ishrat Husain, Matthew J. Burke, Venkat Bhat

**Affiliations:** 1 Unity Health Toronto, Canada; 2 NYU Grossman School of Medicine, USA; 3 The University of Adelaide, Australia; 4 University of Alberta, Canada; 5 Toronto Metropolitan University, Canada; 6 Leiden University, Netherlands; 7 University of Ottawa, Canada; 8 University of Toronto, Canada

**Keywords:** hallucinogens, mental disorders, meta-analysis, psychedelics, randomized controlled trial

## Abstract

**Background:**

Psychedelic-assisted psychotherapy has shown potential for psychiatric disorders. However, the magnitude of symptom change in control groups remains poorly understood. We aim to evaluate within-group effects in control groups and compare them to treatment groups in psychedelic trials.

**Methods:**

A systematic search was conducted up to 1 July 2025. The study protocol was preregistered in PROSPERO (CRD420251111853).

**Results:**

Fourteen randomized controlled trials (n = 643) were included. Direct between-arm meta-analyses showed greater symptom reductions in treatment compared with control across outcomes, including depressive symptoms (number of study arms [k] = 13; SMD = −0.82; 95% CI = −1.17, −0.47; I^2^ = 60.1%), posttraumatic stress disorder (PTSD) symptoms (k = 10; SMD = −0.89; 95% CI = −1.14, −0.65; I^2^ = 0%), and anxiety symptoms (k = 5; SMD = −0.66; 95% CI = −0.94, −0.38; I^2^ = 0%). Subgroup analyses showed limited evidence that effects differed by placebo type for depressive or PTSD symptoms. Descriptive within-group analyses indicated symptom reductions from baseline in both control and treatment groups, with larger within-group improvements observed in treatment groups across outcomes; notably, larger within-group reductions in PTSD symptoms were observed in inactive placebo groups. Sensitivity analyses showed consistent results.

**Conclusions:**

Control groups in psychedelic trials demonstrated substantial symptom improvement, which may reflect non-specific trial factors (including expectancy and concurrent psychotherapy). These findings emphasize the importance of robust control conditions in psychedelic research and the need for nuanced interpretation of treatment effects.

## Introduction

Mental disorders impose a significant global burden, with an estimated economic impact of 4.7 trillion USD and 125.3 million disability-adjusted life years (DALYs) lost worldwide in 2019 [1, 2]. Common first-line treatments for most mental disorders include psychotherapy and pharmacotherapy [3]. However, research suggests that 30%–40% of individuals do not achieve significant symptom improvement with current treatment options [3–5]. While the combination of psychotherapy and pharmacotherapy can demonstrate greater effectiveness than treatment-as-usual or placebo [6–9], these approaches have notable limitations. Reported effects for pharmacotherapy, psychotherapy, and their combination are generally small to moderate, and meta-analyses frequently identify high publication bias, which may lead to an overestimation of treatment efficacy [6–9].

Recent research has explored the potential of psychedelics as a novel treatment for mental disorders. Preliminary evidence suggests that psychedelic-assisted psychotherapy may alleviate symptoms of various mental disorders, such as posttraumatic stress disorder (PTSD), depression, and anxiety, with generally mild and short-term adverse events (AEs) [10, 11]. Despite growing enthusiasm for psychedelic-assisted psychotherapy, the field is constrained by small, homogeneous samples and methodological challenges that limit the interpretability and generalizability of findings [11–16]. A particularly underexamined issue is how placebo and non-specific effects are handled in clinical trials involving psychedelics – interventions that are deeply experiential, highly suggestible, and often embedded in intensive psychotherapeutic frameworks. Randomized controlled trials (RCTs) remain the gold standard for evaluating new treatments [17, 18], but they are ill-equipped to disentangle the overlapping effects of expectancy, therapeutic alliance, and psychotherapeutic context – factors that are especially potent in psychedelic studies. These trials frequently rely on active or low-dose psychedelic placebos combined with extensive psychological support, making it difficult to isolate the drug’s specific therapeutic contribution. Moreover, the powerful subjective effects of psychedelics often result in functional unblinding, further amplifying expectancy and undermining internal validity [19–21].

Unlike typical pharmacotherapy trials, where placebo groups receive inert pills with minimal interaction, control groups in psychedelic trials often receive a credible but subtherapeutic agent along with full psychotherapy protocols. This creates a placebo context rich in therapeutic elements – preparation, guided dosing, and integration – that may independently improve outcomes. Furthermore, due to the inherent difficulties in maintaining blinding in psychedelic trials, accurately estimating treatment effects is contingent upon the choice of control condition [22, 23]. However, the field currently lacks a standardized or widely accepted control condition. This meta-analysis addresses that critical gap. Our objectives are: (1) to estimate between-group treatment effects on symptom change using direct meta-analyses of change scores; (2) to descriptively quantify within-group symptom changes in control groups and treatment groups; (3) to exploratorily compare within-control symptom changes across different control types (e.g., inactive vs low-dose/active placebo) where data permit, including comparisons of within-control change (and, secondarily, whether between-group effects differ by placebo type). By doing so, we aim to clarify how much improvement in psychedelic trials may be attributable to non-drug factors, a question central to interpreting efficacy and designing future studies.

## Methods

This meta-analysis was prepared in accordance with the Preferred Reporting Items for Systematic Reviews and Meta-Analysis (PRISMA) guidelines [24] and was registered in PROSPERO (CRD420251111853).

### Search strategy

A comprehensive search of the literature was conducted on 1 July 2025 through OVID (MEDLINE, Embase, and APA PsychInfo) and PubMed to identify studies evaluating the use of psychedelics for the treatment of psychiatric disorders. The search strategy included the following psychedelics: psilocybin, lysergic acid diethylamide (LSD), dimethyltryptamine (DMT), 3,4-Methylenedioxymethamphetamine (MDMA), and ayahuasca. Psychedelics that targeted NMDA receptors, such as ketamine, were excluded due to their distinct, non-serotonergic mechanisms of action. Search terms included anxiety and related disorders, depression, bipolar disorder, schizophrenia, PTSD, and eating disorders. Results were filtered to RCTs. No language or date restrictions were applied.

### Inclusion/exclusion criteria and screening

Studies underwent a two-stage screening process: first-level screening, which included an assessment based on title and abstract, followed by second-level screening, which involved a full-text assessment. Eligible studies were peer-reviewed RCTs that (1) involved participants with a diagnosed mental disorder, and (2) investigated the efficacy of psychedelics or psychedelic-assisted therapy versus a placebo control condition (active or inactive). Exclusion criteria were as follows: (1) concurrent use of any other forms of pharmacotherapy or drug treatment (e.g., antidepressants); (2) non-human studies; and (3) non-English publications. Screening was performed by two independent reviewers (RS and SM). Conflicts between the two reviewers were resolved through discussion and, when necessary, consultation with VB.

### Data extraction

The following data on study design and patient demographics were extracted by two independent reviewers (RSSM): country of origin, mental disorder of patient population, diagnostic assessment method (e.g., Diagnostic and Statistical Manual of Mental Disorders [DSM] IV or V), mean age, sex (male or female), and sample size. Regarding treatment, the type of psychedelic, dose, and duration (in weeks), route of administration, type of placebo, and concurrent psychotherapy treatment (if applicable) were extracted. Outcome data comprised scores from validated, disorder-specific instruments at all reported assessment time points.

### Quality assessment

Two independent reviewers (RS and SM) assessed the quality of all included studies using the Cochrane Risk of Bias tool [25] (Supplementary Table 1).

### Meta-analysis

All analyses were conducted using R software (version 4.4.0). For each outcome (depressive symptom severity, PTSD symptoms, and anxiety symptom severity), our primary efficacy analysis was a between-group meta-analysis of standardized differences in change scores (treatment vs control). When the mean change was not reported, it was calculated as endpoint mean minus baseline mean. The standard deviation (SD) of the change score was obtained using a predefined hierarchy: (1) reported SD of change; (2) derived from standard error (SE) of change; (3) derived from the 95% confidence interval (CI) of change; or, if none were available, (4) derived from baseline and endpoint SDs assuming a pre–post correlation of r = 0.5. For trials with two treatment arms sharing one control group, the control group sample size was split evenly across comparisons (rounded to the nearest integer), following Cochrane guidance for multi-arm trials. Between-group change-score meta-analyses were conducted using the meta package (version 8.0-2) with the standardized mean difference (Cohen’s d) based on group sample sizes, mean changes, and SDs of change [30].

To contextualize symptom change within psychedelic trials, we conducted secondary within-group meta-analyses separately for treatment and control arms using standardized mean change with change-score standardization (SMCC), which quantifies pre–post changes while accounting for the paired structure of the data [26]. These analyses were intended to describe the magnitude of symptom change occurring within each arm, capturing placebo-associated and other non-pharmacological effects in control groups, and were not designed to estimate treatment efficacy or to isolate a causal placebo effect. Effect sizes were computed using the escalc() and rma() functions from the metafor package (version 4.8-0) [27]. Studies that reported change-score dispersion (SD/SE/CI) were analyzed directly from the change statistics; studies with baseline/endpoint data only used the derived SD change with the same assumed r = 0.5. One study [34] in the control group was excluded from the SMCC meta-analysis due to a very small sample size (n = 2), which led to a non-estimable effect size.

Given anticipated heterogeneity across trials, all meta-analyses used random-effects models. We also conducted sensitivity analyses varying the assumed pre–post correlation used to derive SD of change (r = 0.3, 0.7, and 0.9) to assess robustness [28–31]. In an additional sensitivity analysis restricted to the primary correlation assumption (r = 0.5), we repeated the meta-analyses for depressive and PTSD symptoms after excluding studies rated as having a high risk of bias or some concerns of bias; this analysis was not conducted for anxiety due to an insufficient number of studies. Control conditions were classified as inactive placebo versus active/low-dose placebo, and subgroup analyses by placebo type were performed as exploratory analyses for depressive and PTSD symptoms. Because few studies contributed to each subgroup, subgroup findings should be interpreted cautiously and viewed as exploratory rather than definitive. Statistical heterogeneity was summarized using I^2^ and τ^2^. Effect sizes were interpreted following standard thresholds, where Cohen’s d or SMCC values of 0.2, 0.5, and 0.8 represent small, medium, and large effects, respectively [32]. Heterogeneity across studies was assessed using the I^2^ statistic, with values classified as follows: 0%–40% (not important), 30%–60% (moderate), 50%–90% (substantial), and 75%–100% (considerable) [32]. Potential small-study effects/publication bias were assessed using funnel plots and Egger’s regression test when ≥10 study arms were available for a meta-analysis. For between-group analyses, k reflects the number of comparisons; for within-group analyses, k reflects the number of unique arms contributing data.

## Results

### Search results

**Figure 1.**
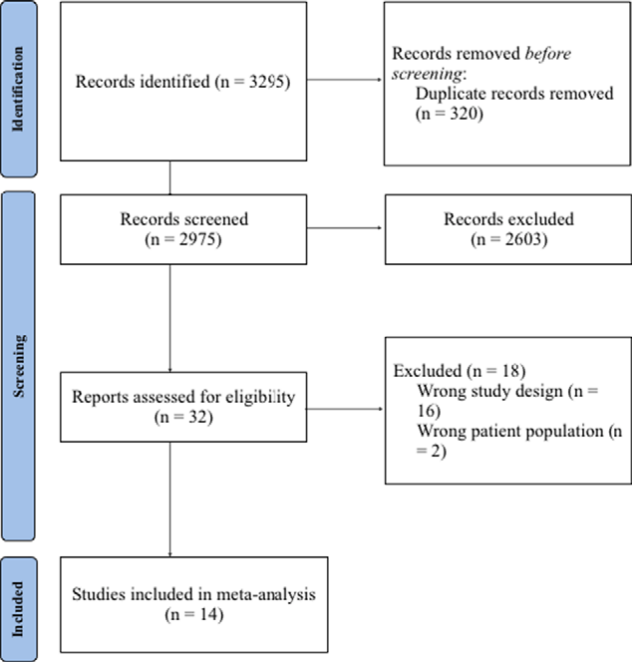
PRISMA flow diagram.

The initial search identified 3295 articles. Duplicates were removed prior to screening (n = 320). A total of 2975 studies underwent first-level screening, and 32 full-text articles were retrieved for second-level assessment. Finally, 14 RCTs (n = 643) met the inclusion criteria and were included in the meta-analysis (Figure 1) [33–46]. Characteristics of included studies are indicated in Table 1.

**Table 1.** Included the characteristics of the studies

Article	Country	Patient Diagnosis	Treatment N (M:F)	Treatment Mean Age (SD)	Placebo N (M:F)	Placebo Mean Age (SD)	Psychedelic Used	Placebo Used	Treatment Dose and Duration	Psychotherapy Protocol
Back et al. [33]	USA	MDD	15 (8:7)	40 (n.s.)	15 (7:8)	36 (n.s.)	Psilocybin	100 mg niacin	25 mg	All sessions were 60–90 minutes. The 2 preparation sessions occurred 8 days and 1 day before the experimental session. Three integration sessions occurred on days 1, 8, and 15.
Bouso et al. [34]	Spain	PTSD	4 (0:4)	37 (8.83)	2 (0:2)	32 (4.24)	MDMA	n.s.	50 mg, 75 mg, 100 mg, 125 mg, or 150 mg Sessions were 6 hours plus another 2 hrs of rest.	Three 90-minute psychotherapy sessions before the experimental session. Three 90-minute psychotherapy sessions after the experimental session. On the following day and the rest with a 5- to 7-day interval.
Goodwin et al. [35]	Czech Republic, Denmark, Germany, Ireland, the Netherlands, Portugal, Spain, the UK, Canada, USA	MDD	10 mg: 75 (34:41) 25 mg: 79 (35:44)	10 mg: 40.6 (12.8) 25 mg: 40.2 (12.2)	79 (43:36)	38.7 (11.7)	Psilocybin	1 mg	Single-dose 25 mg or 10 mg. Administration lasted 6–8 hrs. Non-clinical calming atmosphere. Listened to a music playlist while wearing eyeshades.	Before the session, participants met with a therapist at least 3 times to prepare for the psychedelic experience. After the dosing session, participants received 2 integration sessions.
Holze et al. [36]	Switzerland	Anxiety disorder or clinically significant anxiety associated with a life-threatening illness	20 (11:9)	45 (12)	22 (11:11)	46 (13)	LSD	96% ethanol	2x100μg of LSD in 1 mL of 96% ethanol separated by 6 (±2) weeks. Sessions lasted ~12 hrs. 2-period, random-order, crossover design, with 2 LSD (200 mg) sessions, 2 placebo sessions, and 5 study visits per period	Not specified.
Mitchell et al. [38]	USA, Canada, Israel	PTSD	46 (19:27)	43.5 (12.9)	44 (12:32)	38.3 (10.4)	MDMA	Inactive	10 hr fast. Sessions were 8 hrs. Spaced ~4 weeks apart. 1st Session: 80 mg followed by a supplemental 40 mg 1.5–2.5 hrs after first dose. 2nd + 3rd Session: 120 mg followed by 60 mg 1.5–2.5 hrs after first dose.	Three 90-minute preparatory sessions of therapy. Manualized therapy was conducted in accordance with the MDMA-assisted therapy treatment manual. Each experimental session was followed by three 90-minute integration sessions spaced ~1 week apart. The first occurred the morning after the session, and the remaining occurred over the following 3–4 weeks.
Mitchell et al. [37]	USA, Israel	PTSD	53 (21:32)	38.2 (11)	51 (9:42)	40 (9.6)	MDMA	Inactive	10 hr fast. Sessions were 8 hrs. Spaced ~4 weeks apart. 1st Session: 80 mg followed by a supplemental 40 mg 1.5–2.5 hrs after first dose. 2nd + 3rd Session: 120 mg followed by 60 mg 1.5–2.5 hrs after first dose.	Three 90-minute preparatory sessions of therapy. Manualized therapy was conducted in accordance with the MDMA-assisted therapy treatment manual. Each experimental session was followed by three 90-minute integration sessions spaced ~1 week apart. The first occurred the morning after the session, and the remaining occurred over the following 3–4 weeks.
Mithoefer et al. [39]	USA	PTSD	12 (2:10)	40.2 (7.6)	8 (1:7)	40.4 (7.2)	MDMA	Lactose	Two 8 hrs experimental sessions (125 mg) followed by an overnight stay. An optional supplemental dose of 62.5 mg was administered 2–2.5 hrs after the initial dose if the investigators judged it to be safe and advisable, and the subject agreed.	Two 90-minute preparatory sessions within 6 weeks before the first experimental session. Eight integration sessions. One the morning after each experimental session and three times during the month following each session. Additional integration sessions were permitted if needed. A final integration session occurred 2 months after the second experimental session.
Mithoefer et al. [40]	USA	PTSD	75 mg: 7 (6:1) 125 mg: 12 (8:4)	75 mg: 29.1 (4.9) 125 mg: 40.7 (11.1)	7 (5:2)	32.9 (9.7)	MDMA	30 mg	Two 8-hr experimental sessions spaced 3–5 weeks apart. Initial dose followed 1.5–2 hrs later by an optional supplemental half dose.	Three 90-min preparatory psychotherapy sessions. Three 90-min integration psychotherapy sessions followed experimental sessions.
Oehen et al. [41]	Switzerland	PTSD	8 (1:7)	42.1 (12.8)	4 (1:3)	40 (6.2)	MDMA	25 mg followed by 12.5 mg 2.5 hrs later	125 mg followed by 62.5 mg 2.5 hrs later. Three all-day-long sessions (8-hr sessions plus overnight stay).	Two preparatory sessions. Psychotherapy integration session took place the day after each session, followed by two sessions that were 1 week apart.
Ot’alora et al. [42]	USA	PTSD	100 mg: 9 (3:6) 125 mg: 13 (5:8)	100 mg: 39.6 (9.8) 125 mg: 44.6 (15.4)	6 (1:5)	40 (11.7)	MDMA	40 mg followed by half dose ~90 min later	100 or 125 mg. Two 8-hr sessions spaced 1mo apart. A supplemental half dose was available ~90 minutes after the first dose, if not contraindicated.	Three 90-minute preparatory sessions. Three integration sessions follow each experimental session. One the morning following each session and twice more sometime before the next experimental session. Manualized therapy approach.
Palhano-Fontes et al. [43]	Brazil	MDD	14 (3:11)	39.71 (11.26)	15 (5:10)	44.2 (11.98)	Ayahuasca	Liquid (water, yeast, citric acid, zinc sulfate, and caramel colorant)	Single-dose of 1 mL/kg adjusted to contain 0.36 mg/kg of N, N-DMT lasting ~8 hrs.	Received support throughout the experimental session.
Raison et al. [44]	USA	MDD	51 (27:24)	40.4 (10.9)	53 (25:28)	41.8 (11.7)	Psilocybin	100 mg niacin	25 mg during a 7–10 hr dosing session.	6–8 hrs of preparatory sessions and 4 hours of post-dose integration sessions.
von Rotz et al. [45]	Switzerland	MDD	26 (10:16)	37.6 (10.9)	26 (9:17)	35.9 (9.8)	Psilocybin	Pure mannitol	0.215 mg/kg body weight	Two 60 mins preparatory visits, 4–6, and 1 day before psilocybin/placebo administration. 6 hrs during the administration day, and three 60-mins integration visits, 2, 8, and 14 days after intervention.
Wolfson et al. [46]	USA	Anxiety and/or mood disorders + life-threatening cancer	13 (3:10)	55.5 (7)	5 (1:4)	53.2 (10.5)	MDMA	Lactose	Two day-long experimental sessions of 125 mg followed by an optional supplemental dose of 62.5 mg, 90 to 150 min later.	Three preparatory sessions. Three integration sessions after each experimental session. All psychotherapy sessions were 60- to 90-min long.

Note: Initially 30 mg, but after two of the first three participants discontinued due to one vomiting shortly after administration and the other due to personal reasons, it was lowered to 22 mg. Abbreviations: MDD, Major depressive disorder; MDMA, 3,4-ethylenedioxymethamphetamine; PTSD, Post-traumatic stress disorder; UK, United Kingdom; USA, United States of America.

### Quality assessment

Across the 14 included trials, the risk of bias was predominantly low. Ten studies were low risk overall, with low risk across all five domains. Three studies were rated as having some concerns overall: Holze et al. [36] and Wolfson et al. [46], due to some concerns regarding deviations from intended interventions, and von Rotz et al. [45] due to some concerns in outcome measurement, while all other domains in these studies were low risk. One study, Bouso et al. [34], was assessed as high overall risk of bias, driven by high risk due to deviations from intended interventions, with additional concerns for outcome measurement and selective reporting, despite low risk for the randomization process and missing outcome data.

### Meta-analysis results

#### Between-group comparison: Direct meta-analysis of change scores

**Figure 2.**
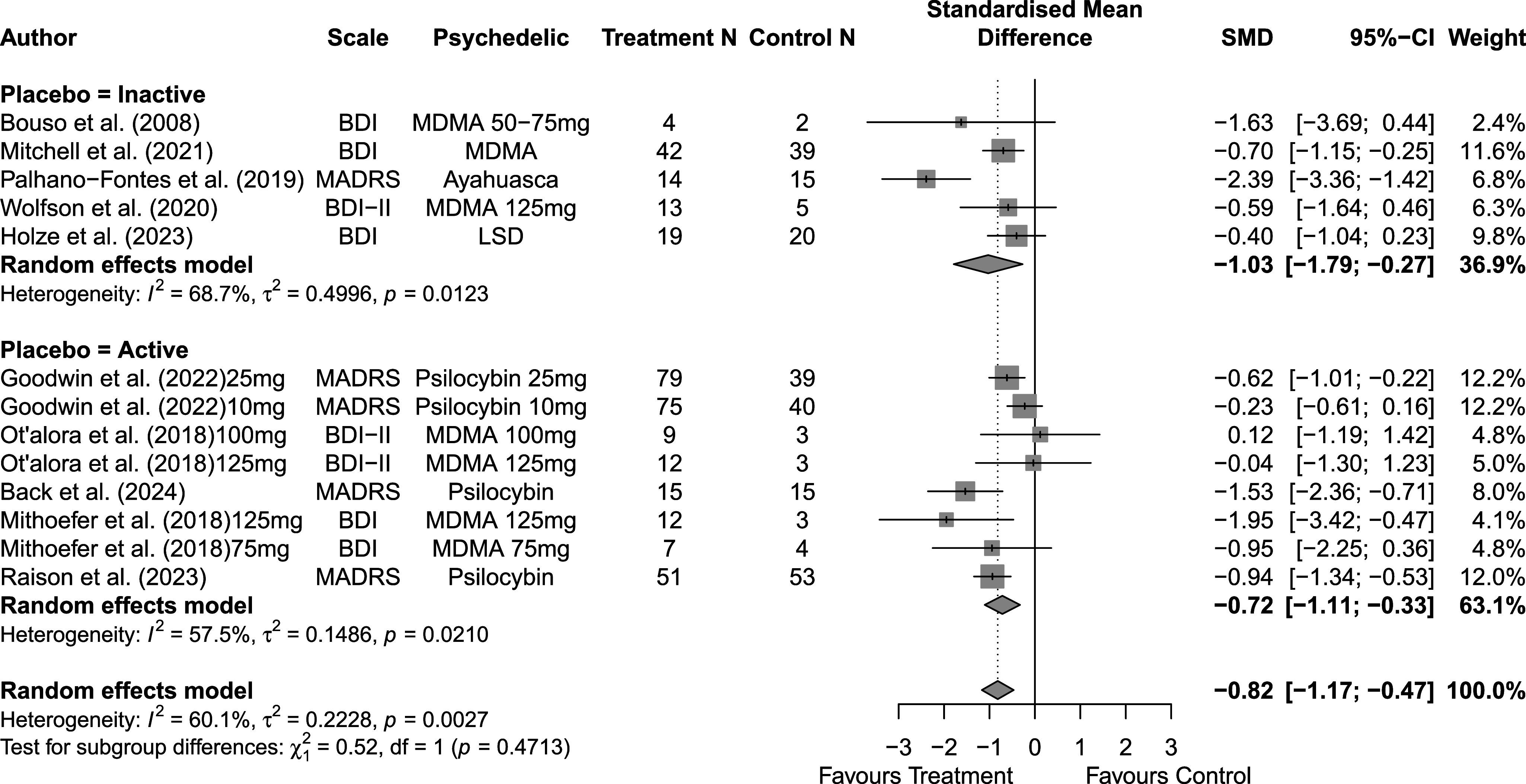
Forest plot of direct between-arm comparisons for depressive symptom change, subgroup by placebo type.

**Figure 3.**
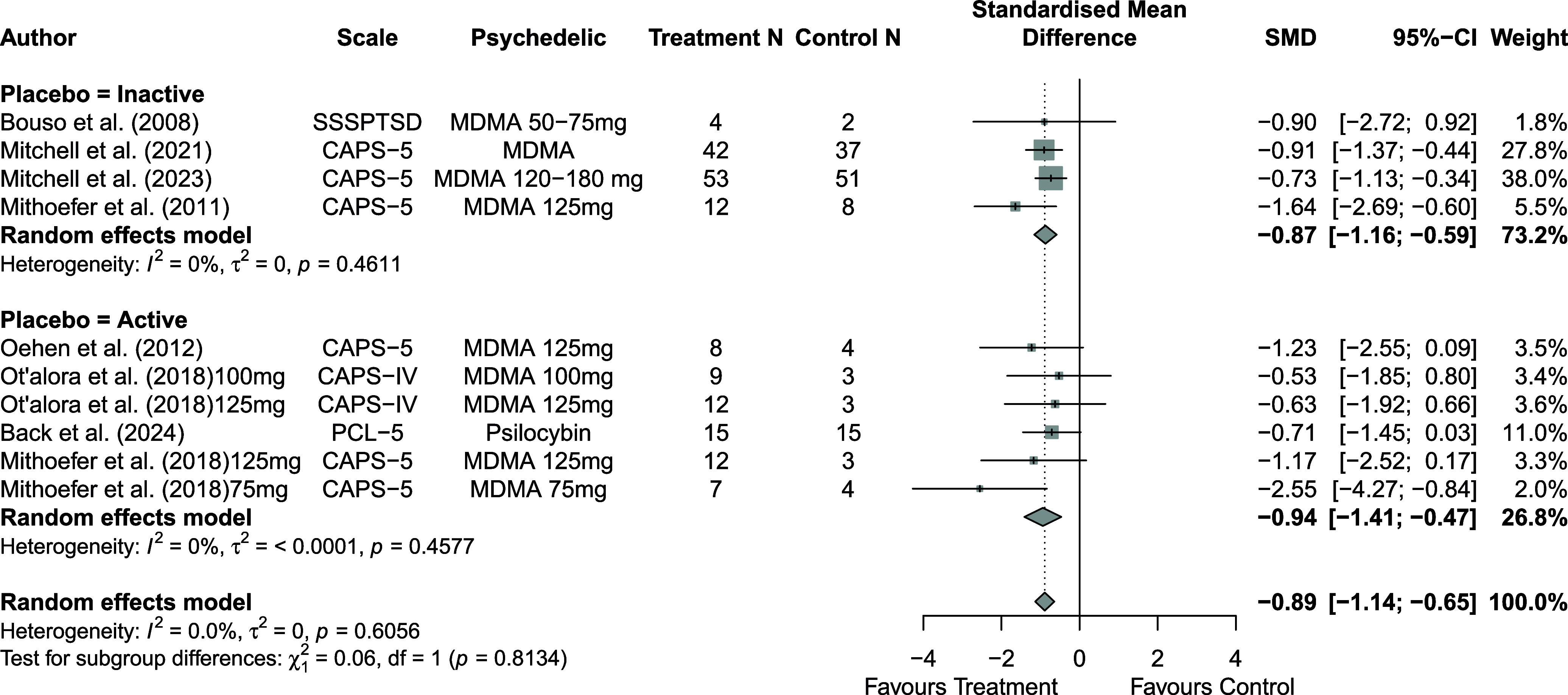
Forest plot of direct between-arm comparisons for PTSD symptom change, subgroup by placebo type.

**Figure 4.**
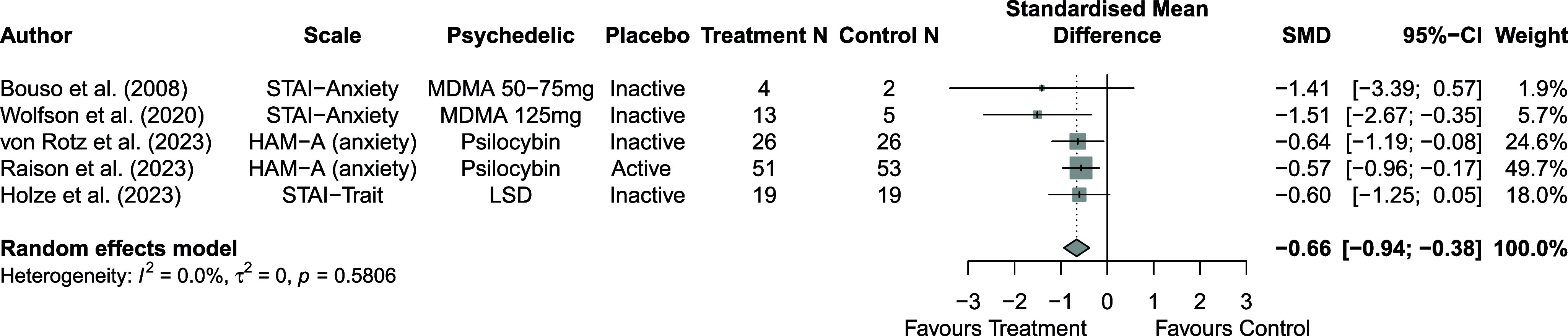
Forest plot of direct between-arm comparisons for anxiety symptom change.

Across 14 trials comprising 17 arm comparisons, eight trials used inactive placebos, while six used active or low-dose placebos. Figures 2–4 present the direct between-arm meta-analyses of change scores for depressive symptoms, PTSD symptoms, and anxiety symptoms. The pooled effect favored treatment across all outcomes, showing large reductions in depressive symptoms (k = 13; SMD = −0.82; 95% CI = −1.17, −0.47; I^2^ = 60.1%) and PTSD symptoms (k = 10; SMD = −0.89; 95% CI = −1.14, −0.65; I^2^ = 0%), and medium reductions in anxiety symptoms (k = 5; SMD = −0.66; 95% CI = −0.94, −0.38; I^2^ = 0%) compared with control. Subgroup analyses did not indicate that effects differed by placebo type for depressive symptoms (inactive k = 5 versus active k = 8; subgroup differences p = 0.47) or PTSD symptoms (inactive k = 4 versus active k = 6; subgroup differences p = 0.81). Funnel plots and Egger’s tests provided limited evidence of small-study effects for depressive symptoms (p = 0.24; Supplementary Figure 1S) and PTSD symptoms (p = 0.15; Supplementary Figure 2S). Sensitivity analyses are presented in Supplementary Table 2S; varying the assumed within-study correlation (r) from 0.3 to 0.9 changed the magnitude of pooled effects (generally becoming larger in absolute value at higher r), while the overall direction remained consistent with greater symptom reduction in the treatment condition. The second sensitivity analysis excluding studies with high or some concerns of bias showed similar results for depressive symptoms (k = 10; SMD = −0.88; 95% CI = −1.31, −0.44; I^2^ = 68.3%; Supplementary Figure 6aS) and PTSD symptoms (k = 9; SMD = −0.89; 95% CI = −1.14, −0.64; I^2^ = 0%; Supplementary Figure 7aS).

#### Within-group effects

Supplementary Figures 3S–5S present within-group meta-analyses of SMCC across outcomes in control and treatment groups. In control groups, pooled estimates indicated medium-to-large symptom reductions across outcomes, including depressive symptoms (k = 9; SMCC = −0.58; 95% CI = −0.77, −0.38; I^2^ = 34%), PTSD symptoms (k = 7; SMCC = −0.75; 95% CI = −1.10, −0.39; I^2^ = 58.2%), and anxiety symptoms (k = 4; SMCC = −0.51; 95% CI = −0.71, −0.30; I^2^ = 0%). Subgroup analyses by placebo type showed no differences for depressive symptoms (inactive k = 4 versus active k = 5; subgroup differences p = 0.70), while larger within-group reductions in PTSD symptoms were observed in the inactive placebo group (SMCC = −1.15 vs. −0.37; subgroup differences p < 0.001).

In treatment groups, within-group analyses showed larger symptom reductions across outcomes, including depressive symptoms (k = 13; SMCC = −1.30; 95% CI = −1.69, −0.92; I^2^ = 84.1%), PTSD symptoms (k = 10; SMCC = −1.47; 95% CI = −1.86, −1.08; I^2^ = 61.7%), and anxiety symptoms (k = 5; SMCC = −1.16; 95% CI = −1.40, −0.92; I^2^ = 0%). Funnel plot inspection (Supplementary Figure 3cS) suggested some asymmetry, and Egger’s test indicated evidence of small-study effects (p = 0.005). Sensitivity analyses varying the assumed within-study correlation (r = 0.3–0.9; Supplementary Tables 3aS–3bS) showed that effect magnitudes changed as expected, while the direction of within-group change remained consistent across outcomes. In a separate sensitivity analysis excluding studies with a high risk of bias or some concerns of bias (Supplementary Figures 6S–7S; conducted under the primary r = 0.5 assumption), pooled effects for depressive symptoms and PTSD symptoms remained consistent.

## Discussion

Our results demonstrated that between-group meta-analyses of change scores consistently favored treatment for depressive symptoms, PTSD symptoms, and anxiety symptoms, with larger benefits for depression and PTSD and more moderate benefits for anxiety, and no meaningful differences by placebo type. Within-group analyses showed symptom reductions in control conditions but larger improvements in treatment groups; placebo type did not materially affect within-group depression changes, whereas control-group PTSD improvements appeared greater with inactive placebo. Small-study effects were limited for between-group findings but suggested in treatment-group within-group analyses, and sensitivity analyses varying the within-study correlation changed effect sizes but not the direction of effects.

The magnitude of symptom reduction observed in control groups is clinically relevant but warrants cautious interpretation. The within-control changes in the present synthesis appear more modest than the “large” placebo responses reported in some broader overviews of mental health trials [47], although direct comparison is limited by differences in sampled populations, concomitant interventions (including psychotherapy), follow-up intervals, and effect-size calculations. In psychedelic-assisted psychotherapy trials, both treatment and control arms typically include substantial clinical contact and structured support [33–46], which may contribute to symptomatic improvement independent of psychedelic exposure; accordingly, observed control-arm changes are best interpreted as reflecting overall response under the trial context rather than evidence for any single operative mechanism. A further methodological consideration is that psychedelic trials may be especially susceptible to lessebo effects, attenuated improvement when participants infer assignment to placebo or a sub-therapeutic condition, particularly in settings characterized by strong prior expectations and imperfect masking [48, 49]. Additional non-specific influences may include reactivity to repeated, structured outcome assessments (e.g., trauma-focused interviews in PTSD trials) [50], regression to the mean, and natural symptom fluctuation. Because expectancy and blinding integrity were inconsistently measured and reported across included studies [49], these potential contributors could not be evaluated quantitatively and should be treated as hypotheses rather than causal explanations.

While between-group subgroup analyses did not indicate clear differences by control type, the within-control analyses suggested that PTSD symptom reductions may be larger in trials using inactive controls than in those using active controls, whereas depressive symptoms did not show a clear pattern by control condition. These observations raise important methodological considerations. Low-dose psychedelics and other active control strategies are often used to preserve blinding, yet they may still compromise trial integrity if participants infer allocation based on the presence or absence (or intensity) of expected acute effects, potentially shaping expectancy, engagement, and therapeutic response [51–52]. This issue is directly relevant to the design of the MAPS phase 3 MDMA trials, which used an inactive placebo comparator; the investigators noted that low-dose MDMA had improved blinding in earlier studies but was not selected for phase 3 in part to better isolate drug efficacy and permit cleaner safety comparisons. More broadly, low-dose psychedelics may not be pharmacologically inert: even sub-perceptual or low doses can produce subtle subjective, cognitive, or physiological effects that could influence participant experience and expectations, complicating interpretation of treatment effects. Finally, the absence of consistent control-type differences in many analyses highlights ongoing challenges in this literature, including limited power to detect subgroup effects and the frequent lack of systematic measurement of expectancy and blinding integrity factors that are critical for improving the rigor and interpretability of psychedelic trials.

Our study has several limitations. First, due to the absence of detailed information on expectancy and blinding across studies, we were not able to evaluate the effects of these factors in our analysis. Differences in participant expectations and inadequate blinding may have biased the results, potentially leading to an overestimation of the intervention’s true effects. Second, since the included psychedelic trials involved psychotherapy sessions even in the placebo groups, the magnitude of the group response cannot be accurately estimated, as the effects of therapy are inherently included. These limitations suggest that future research should aim for more consistent reporting, larger sample sizes, better control for expectancy and blinding, and the inclusion of a therapy-alone condition or, ideally, a 2 × 2 design to enhance the validity and applicability of the findings.

In conclusion, this meta-analysis showed that control conditions in psychedelic-assisted psychotherapy trials were associated with meaningful within-group symptom reductions, with moderate-to-large improvements in depressive and PTSD symptoms and moderate improvements in anxiety. Treatment groups consistently demonstrated greater symptom improvement than controls across outcomes, but the magnitude of change observed in control groups is consistent with an important role for non-pharmacological and contextual factors, although these mechanisms could not be evaluated directly in the included trials. In addition, while between-group effects did not appear to differ by control type, the within-control findings suggested larger PTSD improvements under inactive placebo than active control conditions, underscoring how comparator selection may shape outcomes and interpretation. Collectively, these findings emphasize the need for careful design, justification, and transparent reporting of control conditions, alongside routine assessment of blinding integrity and expectancy, so that the incremental benefit attributable to psychedelics can be interpreted within the therapeutic milieu. Future research should prioritize dismantling and head-to-head comparator designs, as well as mechanistic studies, to better disentangle drug-specific effects from psychological and contextual contributors to response.

## Supporting information

10.1192/j.eurpsy.2026.10168.sm001 Meshkat et al. supplementary material Meshkat et al. supplementary material

## Data Availability

This study is a meta-analysis of previously published data and did not involve the collection of individual participant data. Extracted summary data, as well as the study protocol and analysis code, are available from the corresponding author upon reasonable request.

## Supplementary material

The supplementary material for this article can be found at http://doi.org/10.1192/j.eurpsy.2026.10168.
