# Supplementary material for: Magnitude of response in treatment and control groups within psychedelic trials for psychiatric disorders: A meta-analysis
Source: Eur Psychiatry. 2026 Feb 18;69(1):e32. doi: 10.1192/j.eurpsy.2026.10168 (PMC13122533; doi:10.1192/j.eurpsy.2026.10168)
Supplement: Meshkat et al. supplementary material [file S0924933826101680sup001.docx]

**Supplementary Materials**

**Figure 1S**. Funnel plot of direct between-arm comparisons for depressive symptom change.


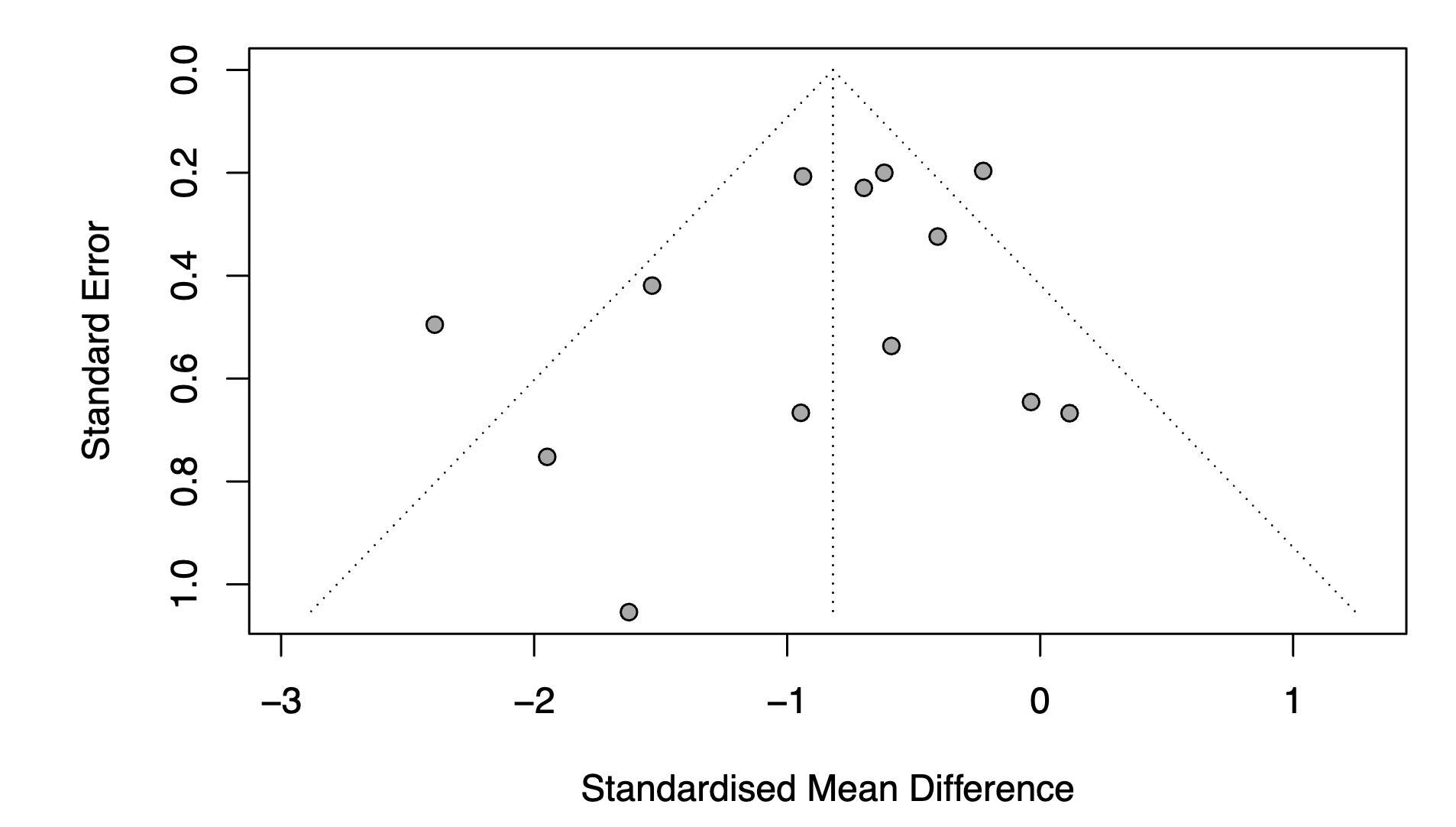


Egger’s p-value is 0.24

**Figure 2S**. Funnel plot of direct between-arm comparisons for PTSD symptom change.


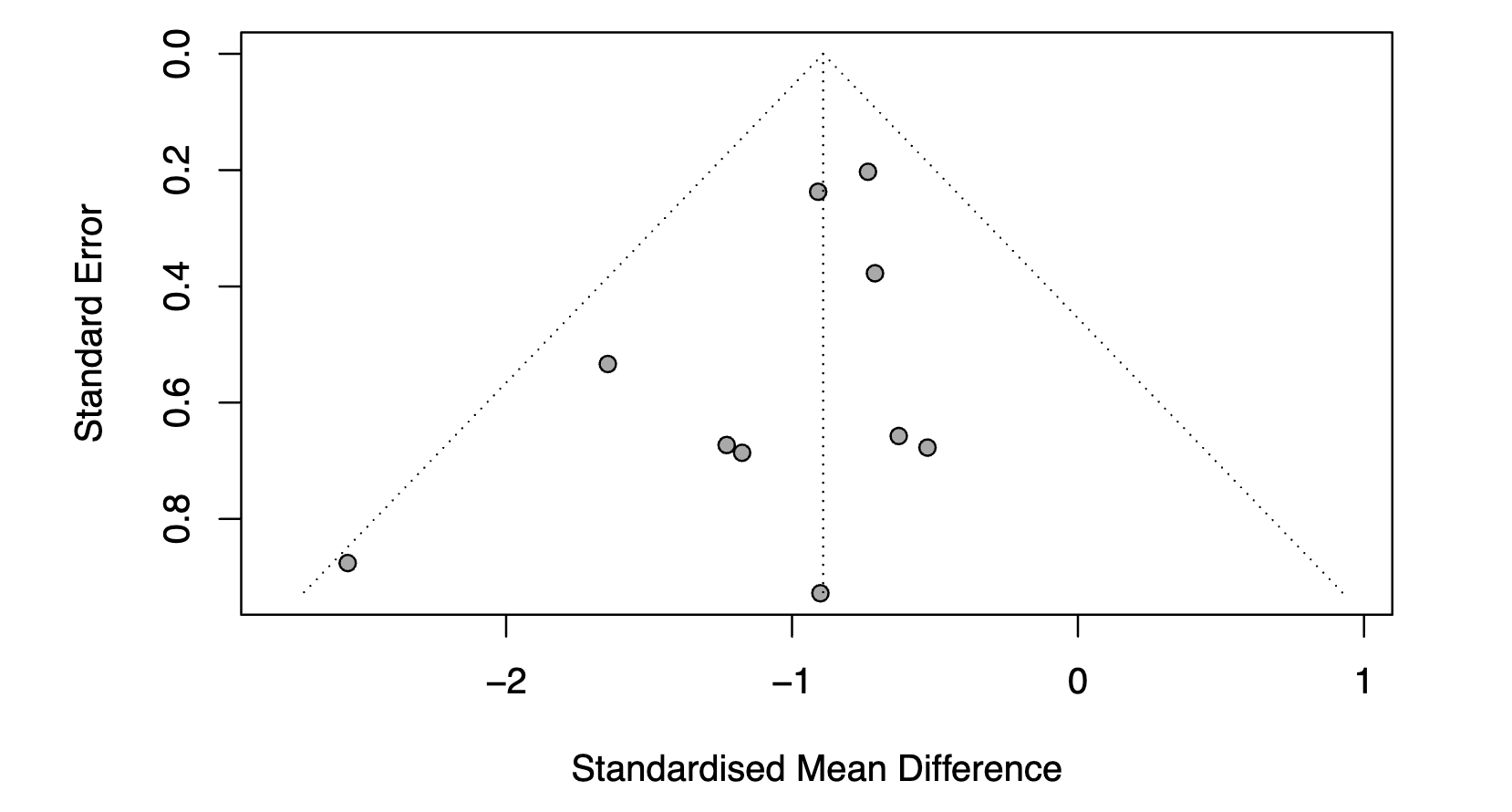


Egger’s p-value is 0.15

**Figure 3aS**. Forest plot of SMCC in depressive symptoms within control group, subgroup by placebo type.


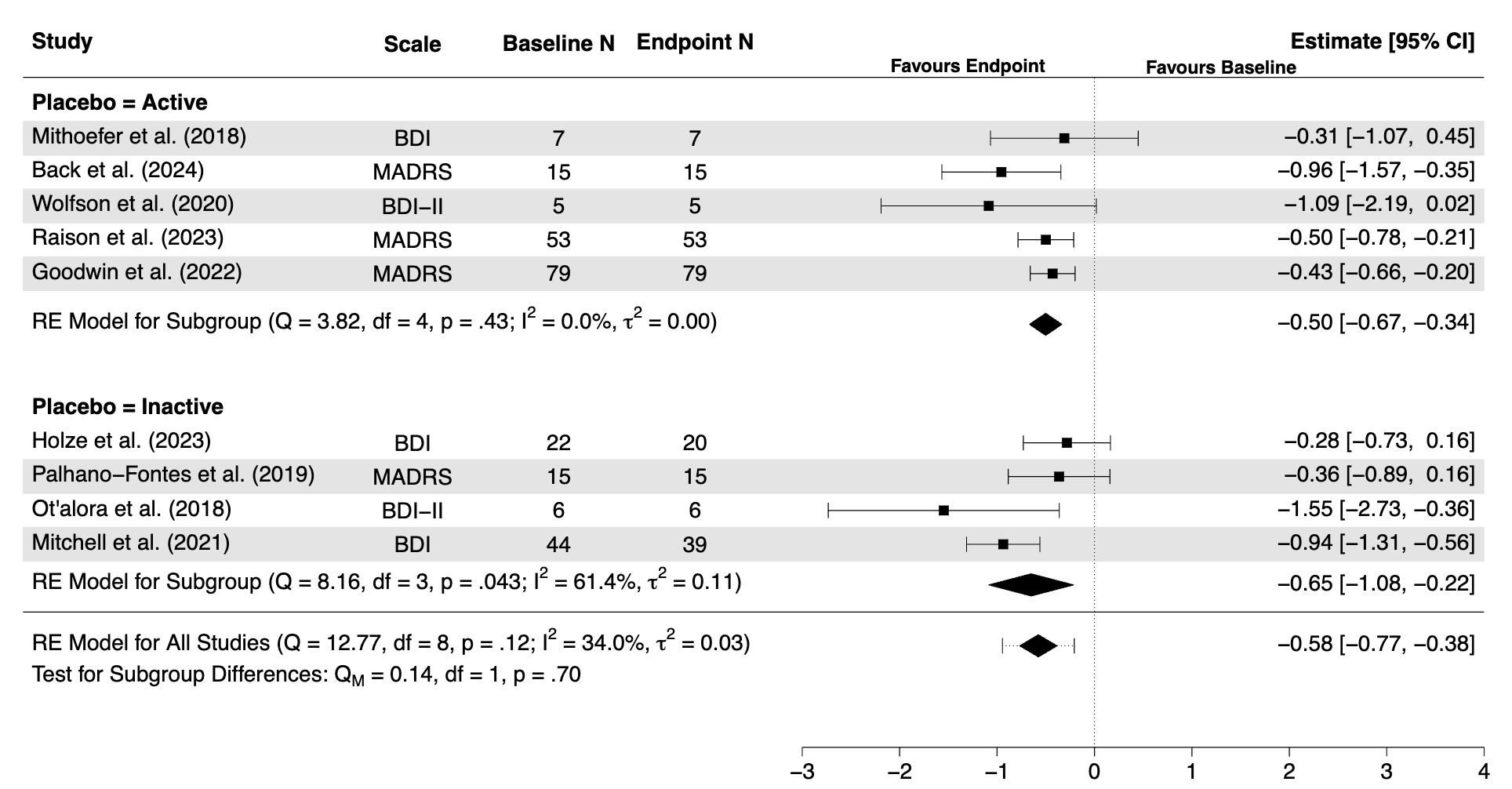


**Figure 3bS**. Forest plot of SMCC in depressive symptoms within treatment groups.


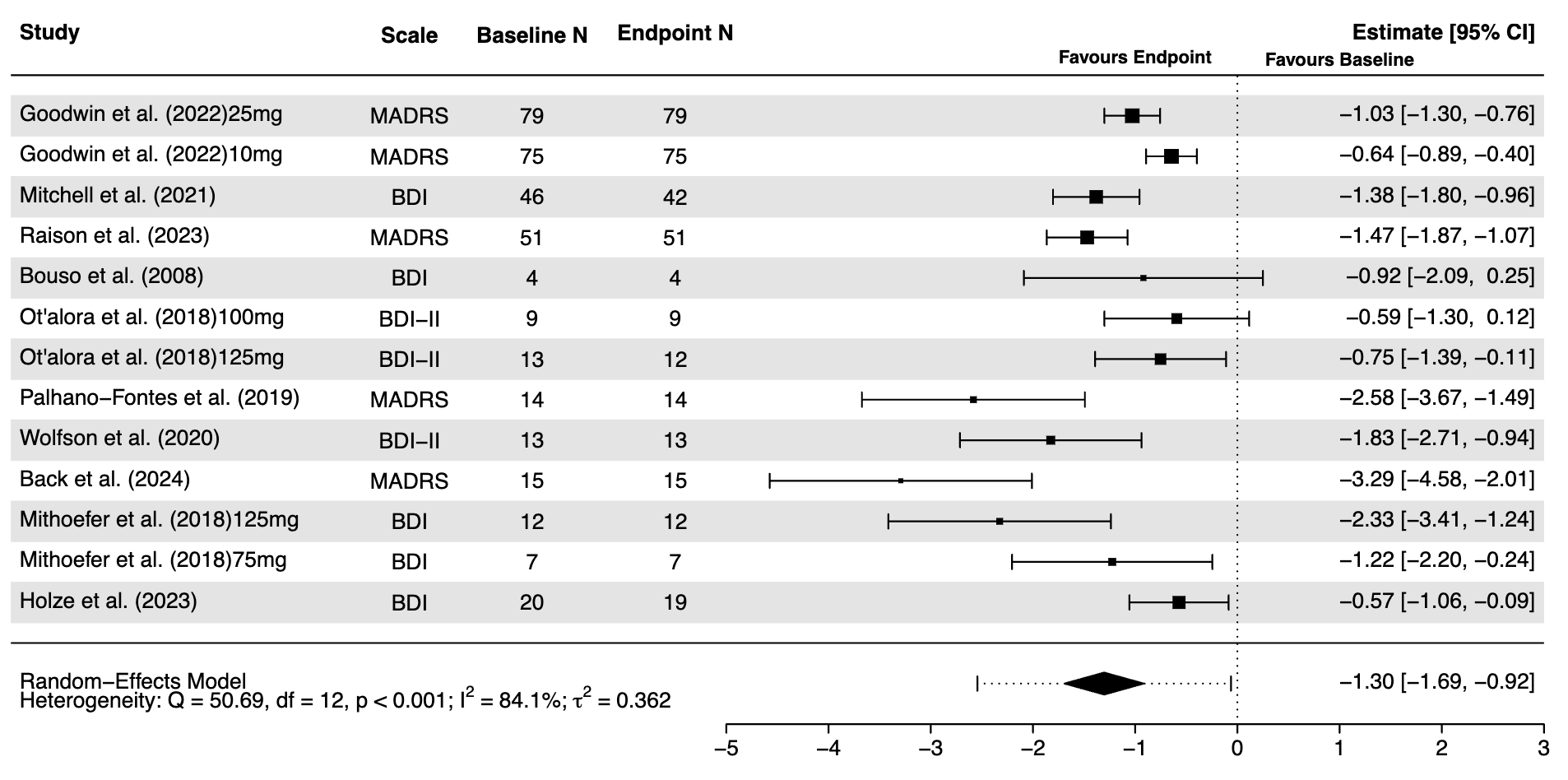


**Figure 3cS**. Funnel plot of within-group change in depressive symptoms in the treatment group.


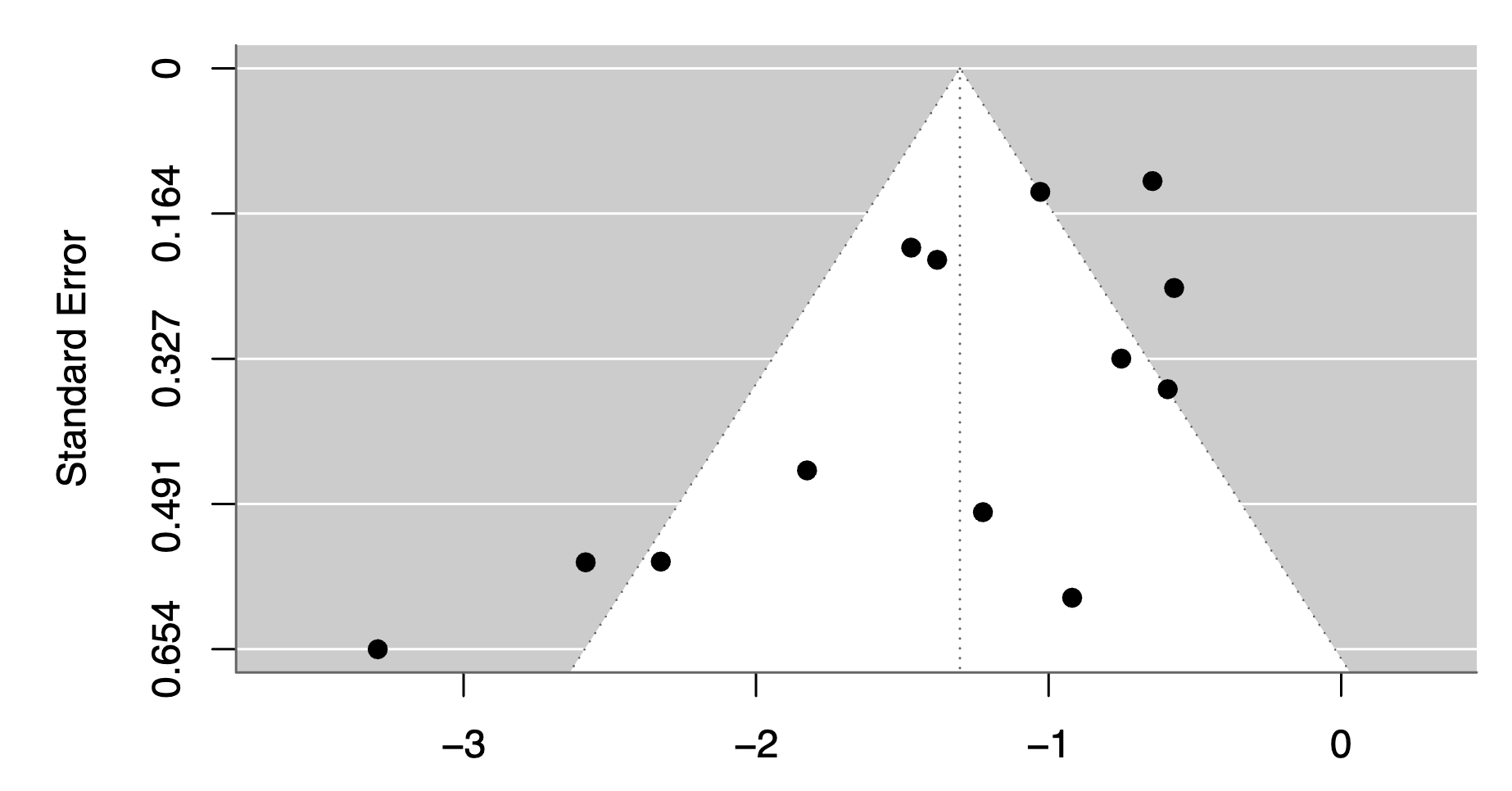


Egger’s p value is 0.005.

**Figure 4aS**. Forest plot of SMCC in PTSD symptoms within control group, subgroup by placebo type.


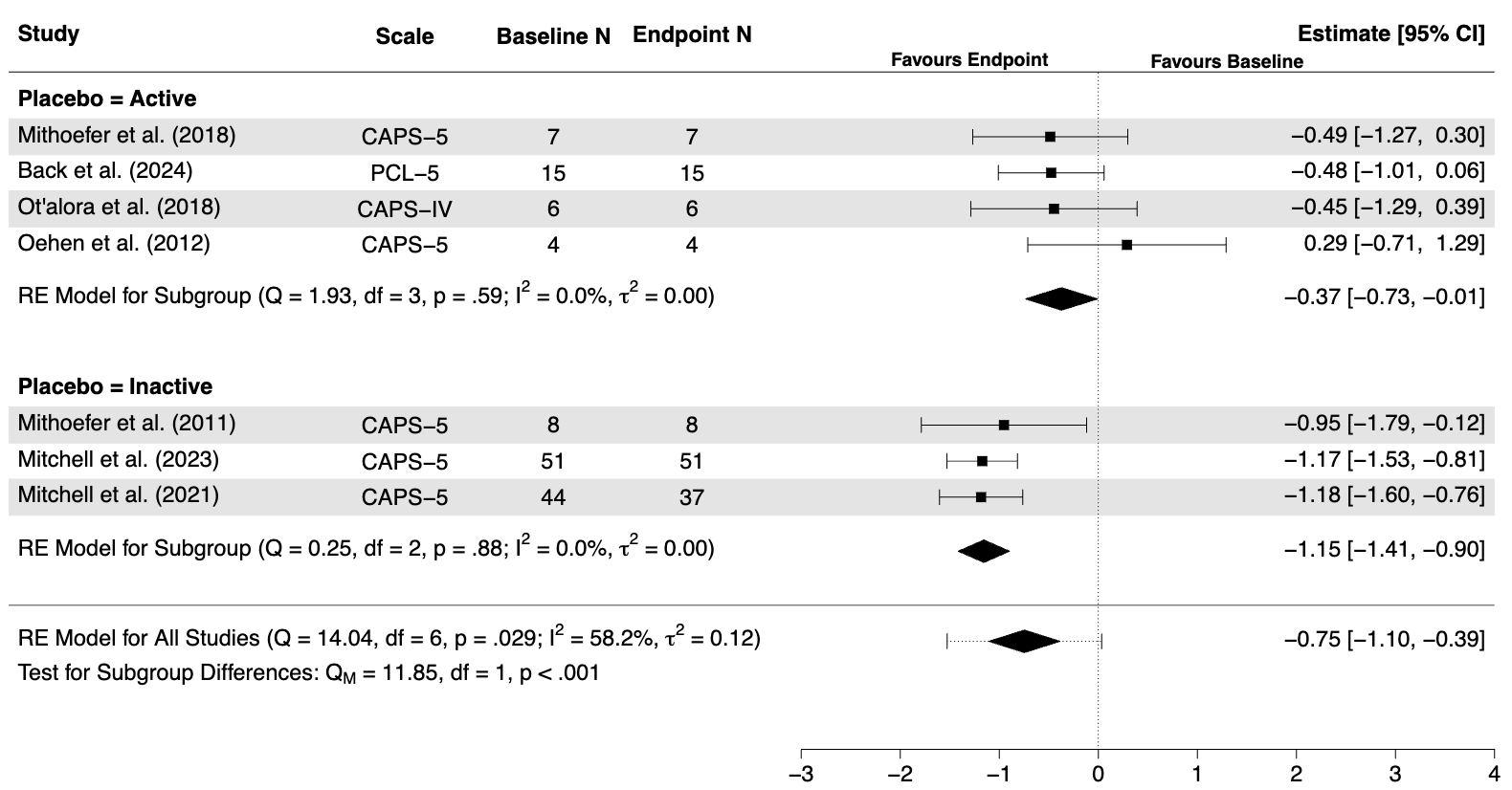


**Figure 4bS**. Forest plot of SMCC in PTSD symptoms within treatment groups.


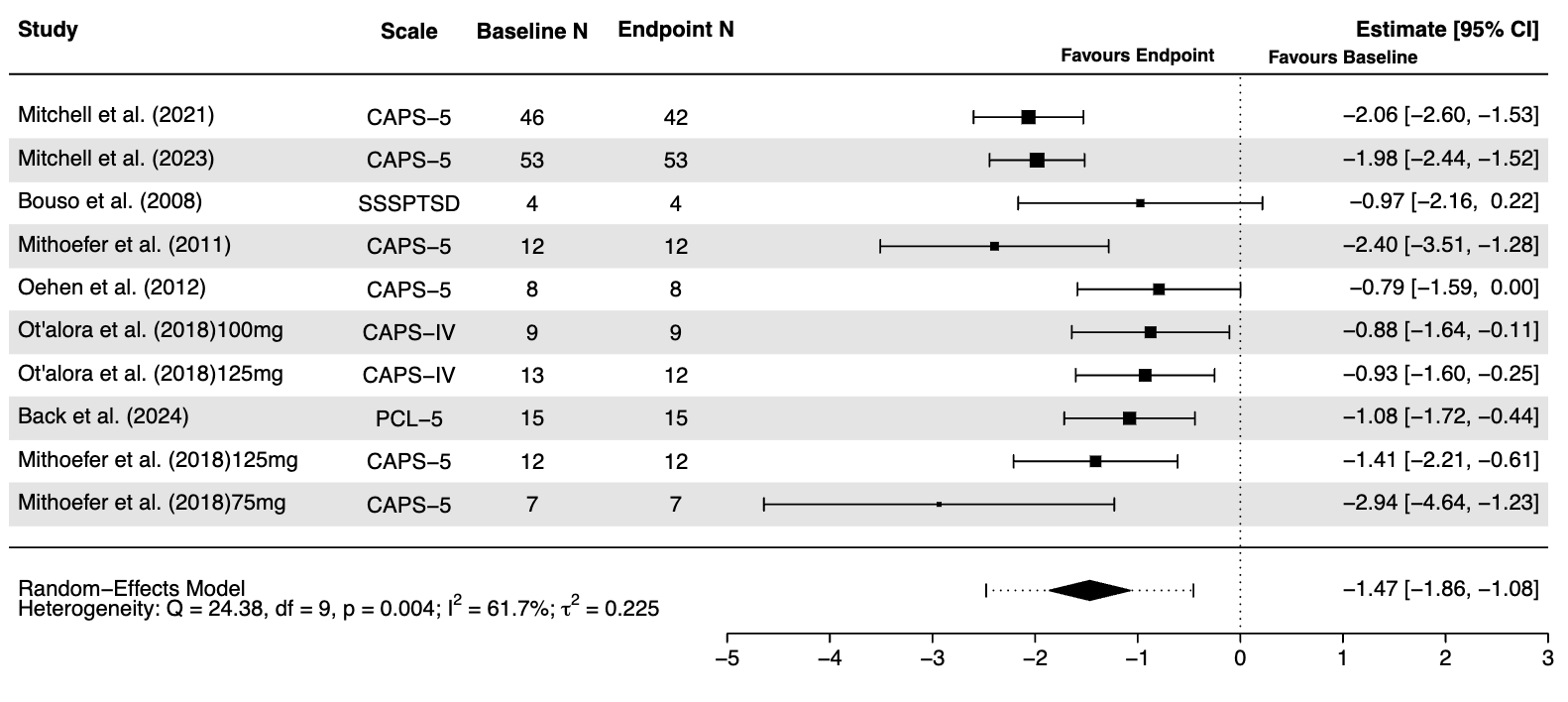


**Figure 4cS**. Funnel plot of within-group change in PTSD symptoms in the treatment group.


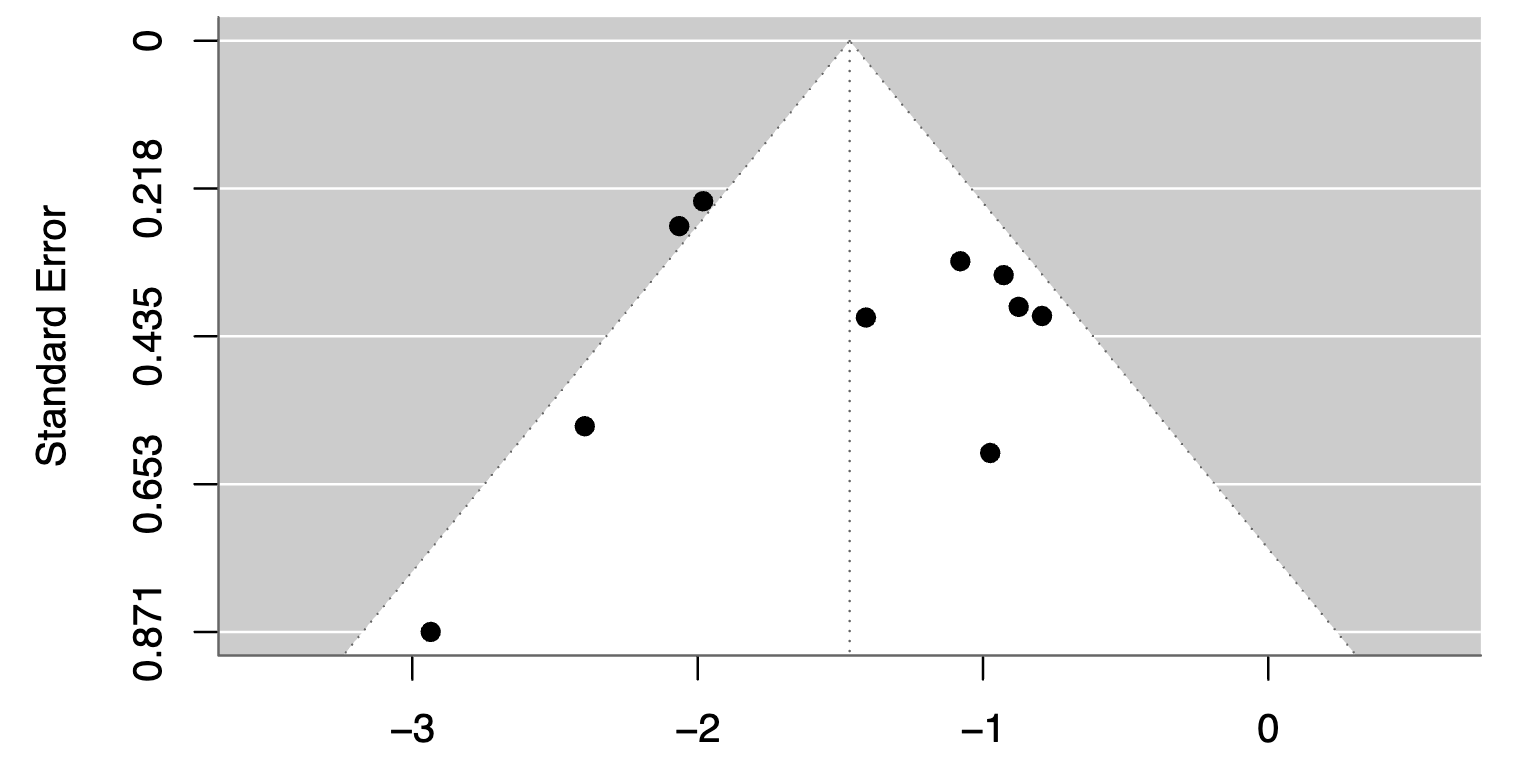


Egger’s p value is 0.51.

**Figure 5aS**. Forest plot of SMCC in anxiety symptoms within control groups.


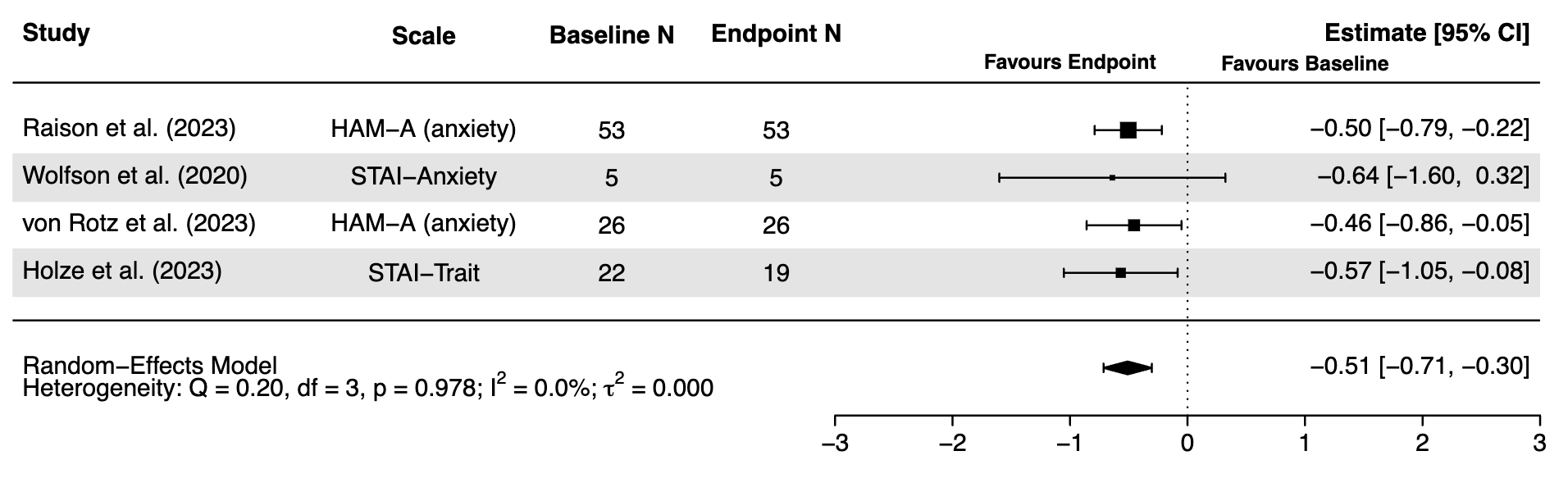


**Figure 5bS**. Forest plot of SMCC in anxiety symptoms within treatment groups.

**
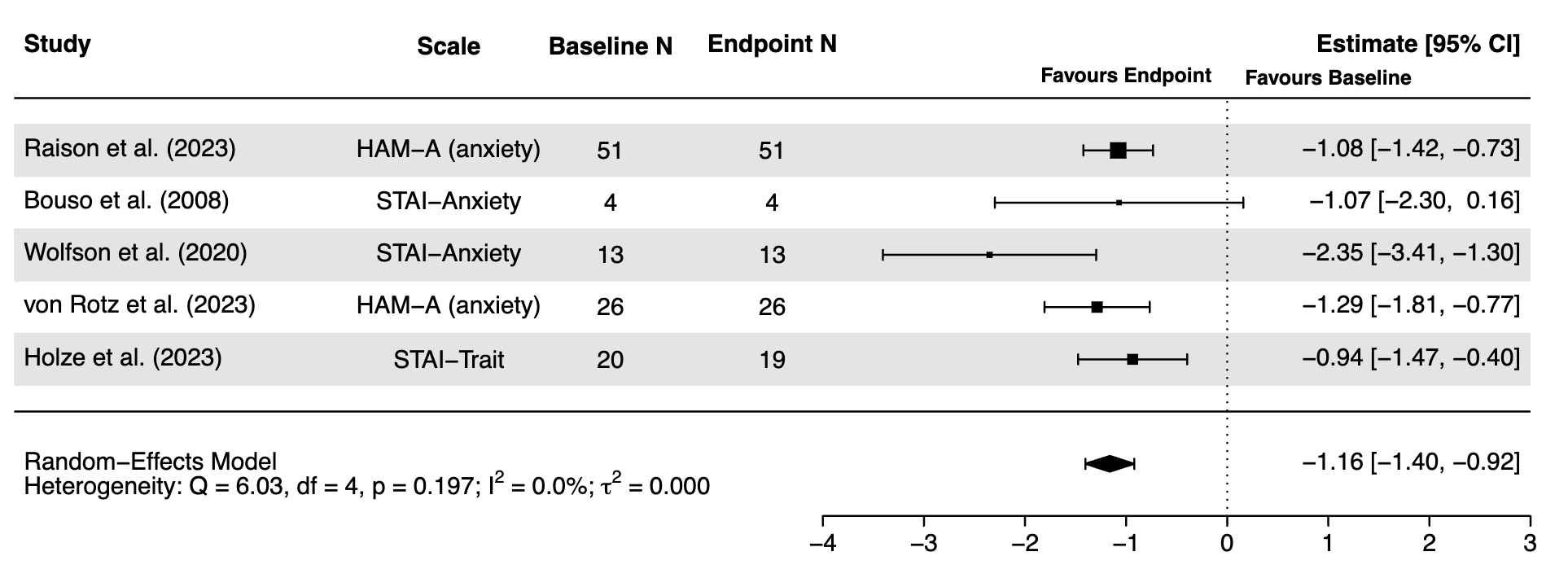
**

**Figure 6aS.** Sensitivity analysis excluding studies with high or some concerns of bias: forest plot of direct between-arm comparisons for depressive symptom change, subgroup by placebo type (k = 10).


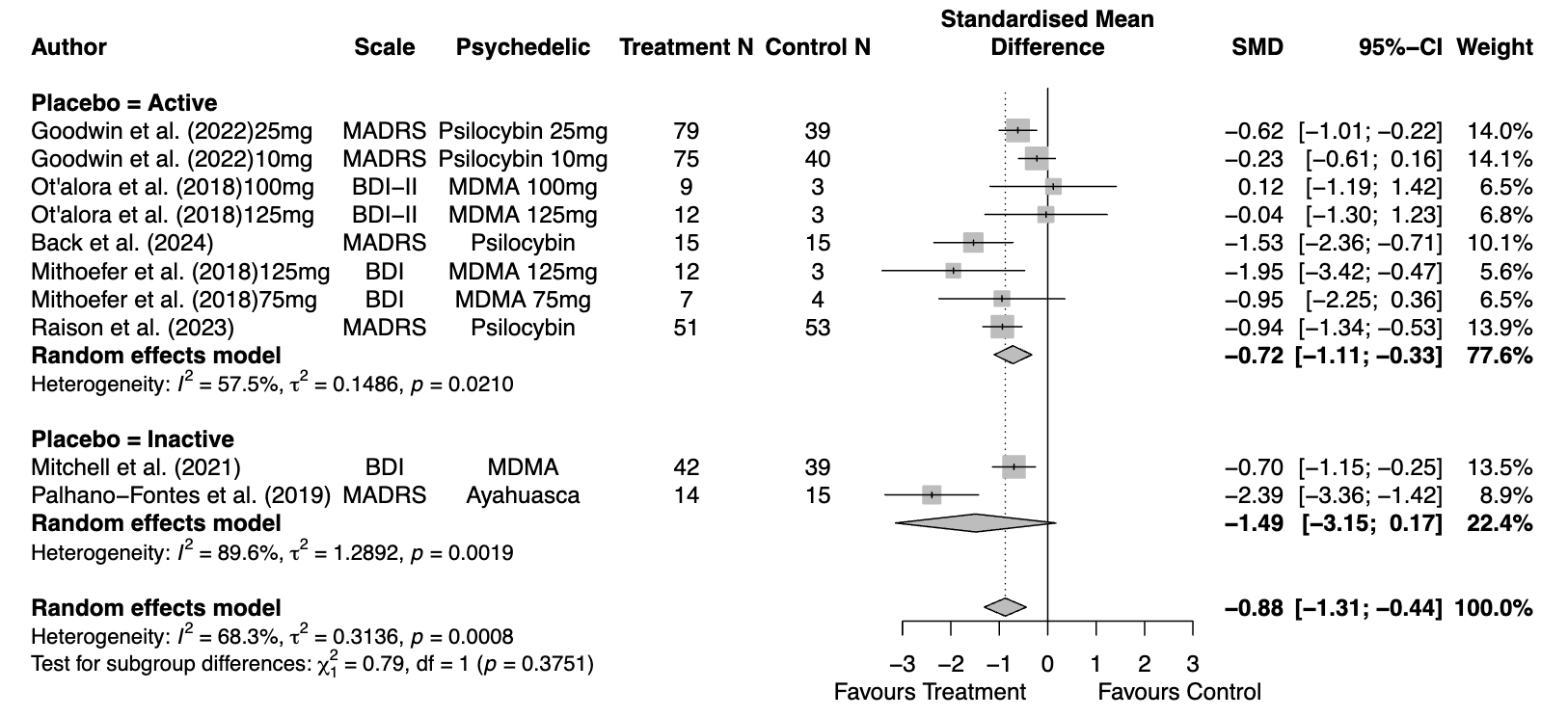


**Figure 6bS**. Sensitivity analysis excluding studies with high or some concerns of bias: funnel plot of direct between-arm comparisons for depressive symptom change (k = 10).


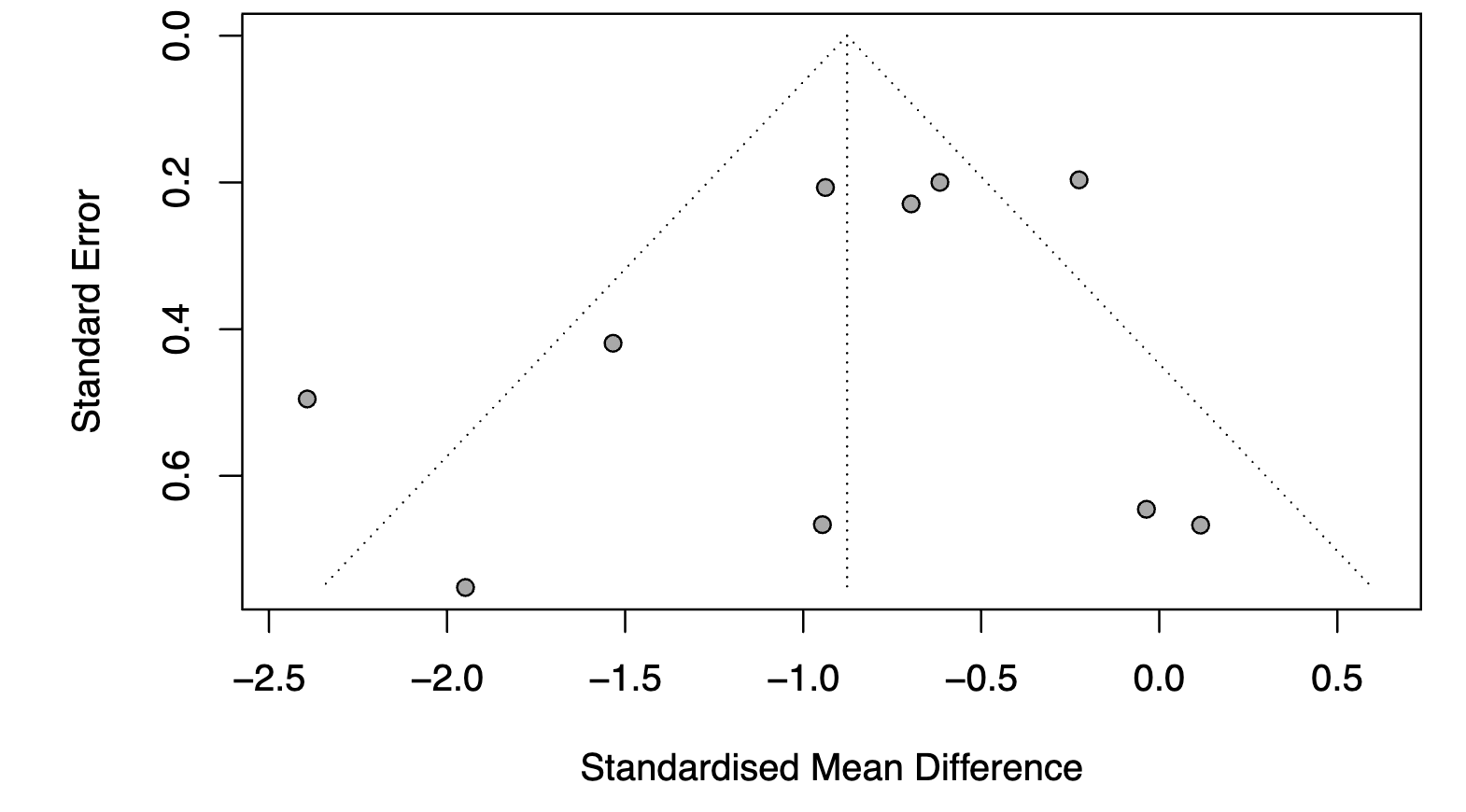


Egger’s p value is 0.29.

**Figure 6cS.** Sensitivity analysis excluding studies with high or some concerns of bias: forest plot of SMCC in depressive symptoms within control group, subgroup by placebo type (k = 7).


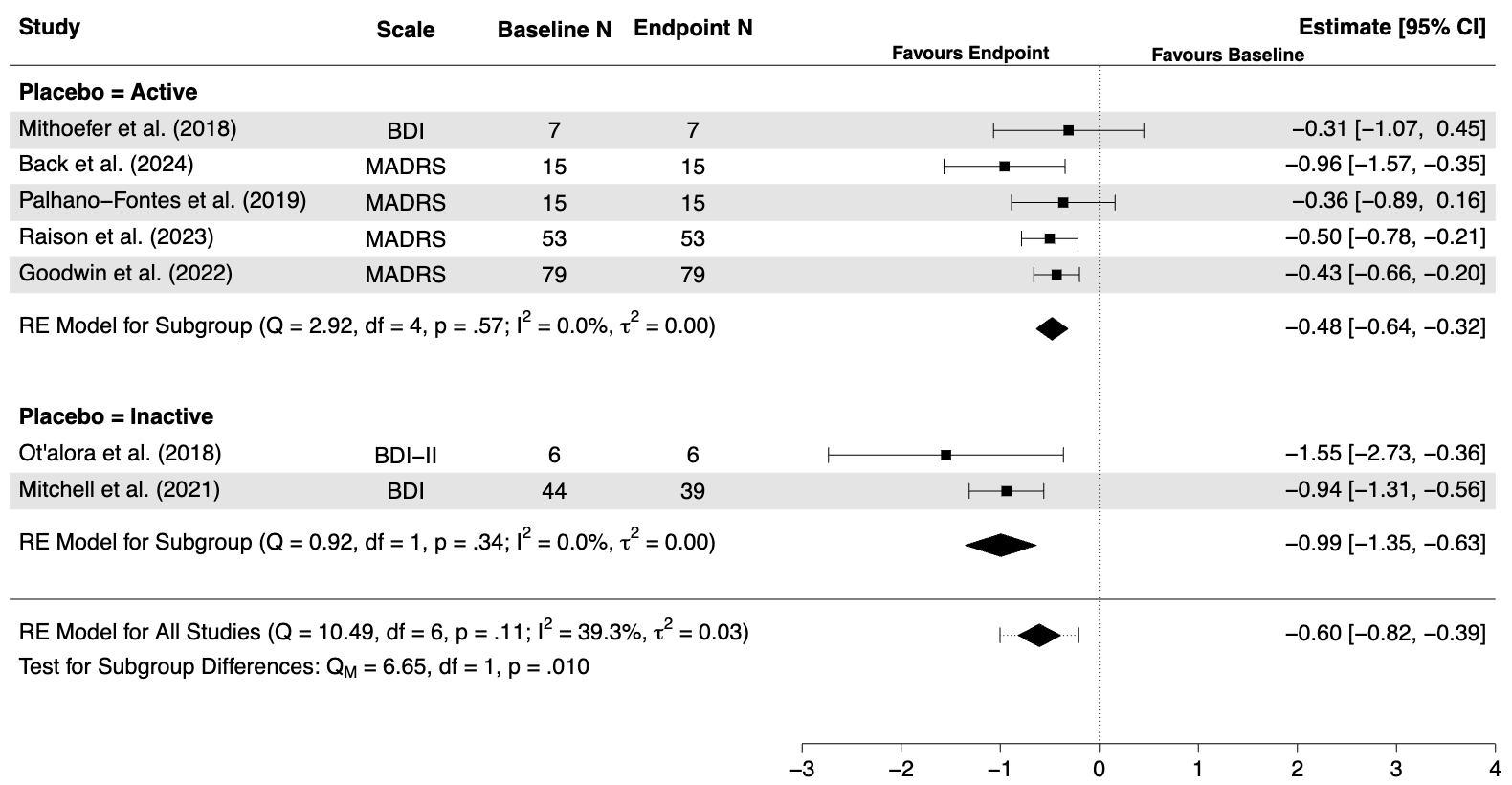


**Figure 6dS.** Sensitivity analysis excluding studies with high or some concerns of bias: forest plot of SMCC in depressive symptoms within the treatment group (k = 10).


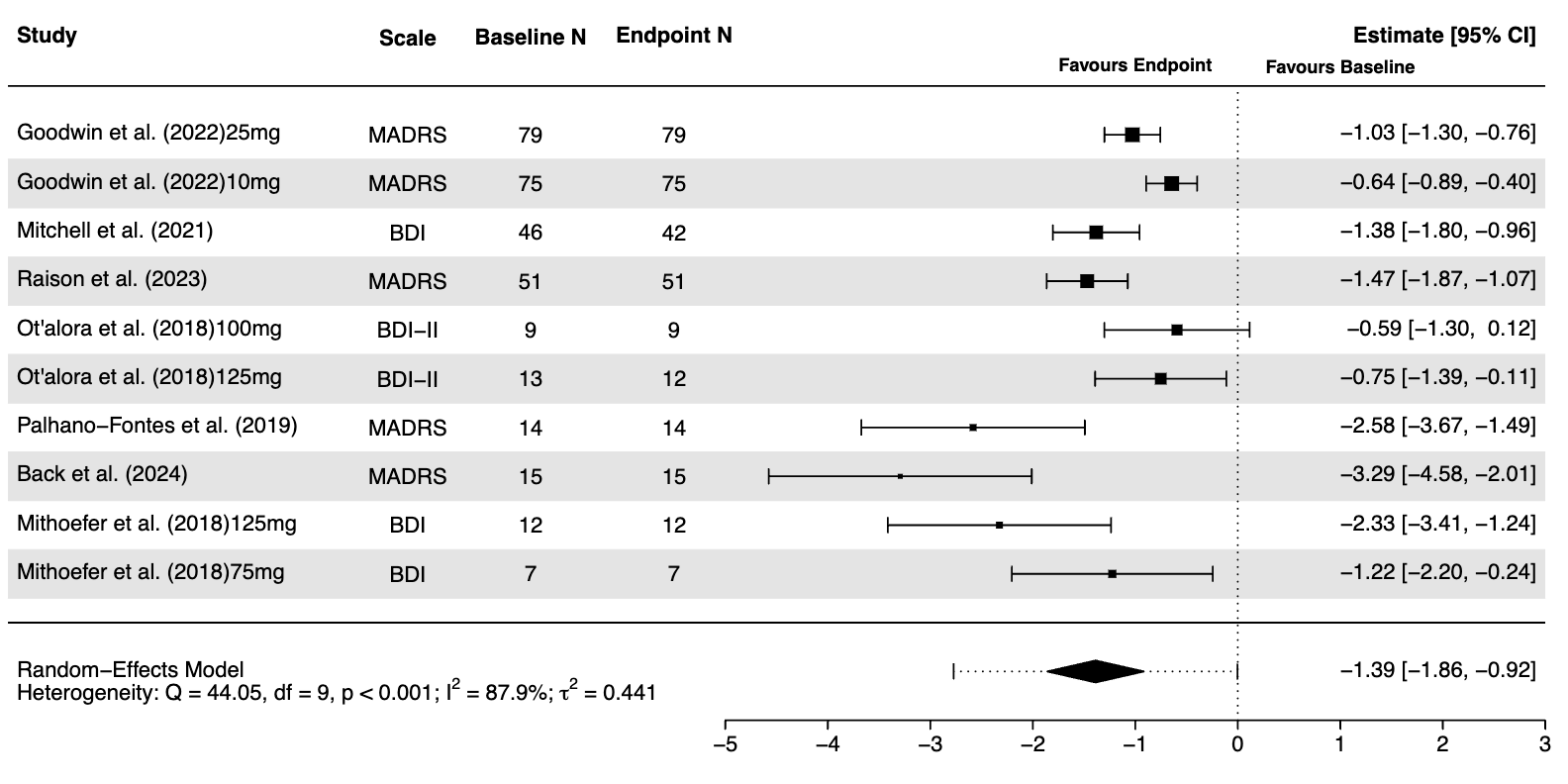


**Figure 7aS.** Sensitivity analysis excluding studies with high or some concerns of bias: forest plot of direct between-arm comparisons for PTSD symptom change, subgroup by placebo type (k = 9).


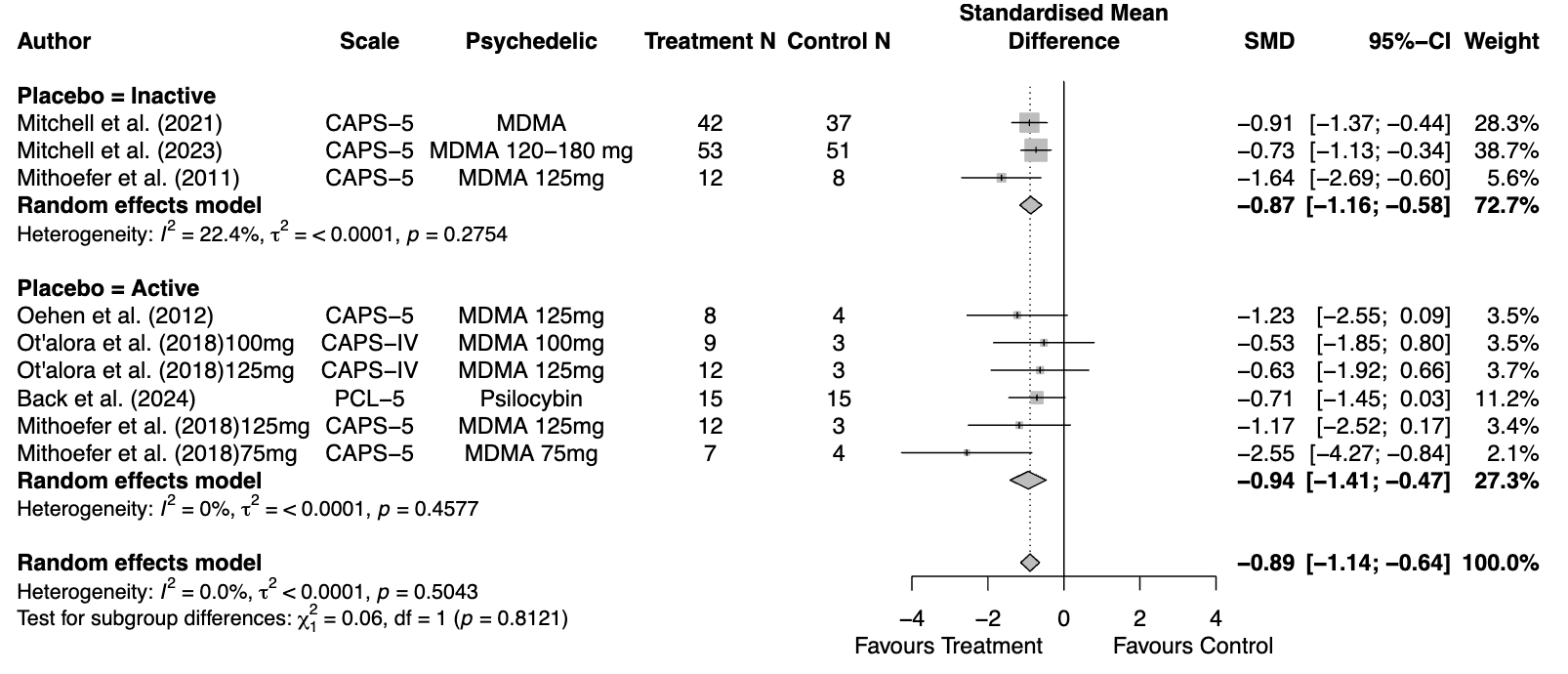


**Figure 7bS.** Sensitivity analysis excluding studies with high or some concerns of bias: forest plot of SMCC in PTSD symptoms within control group, subgroup by placebo type (k = 7).


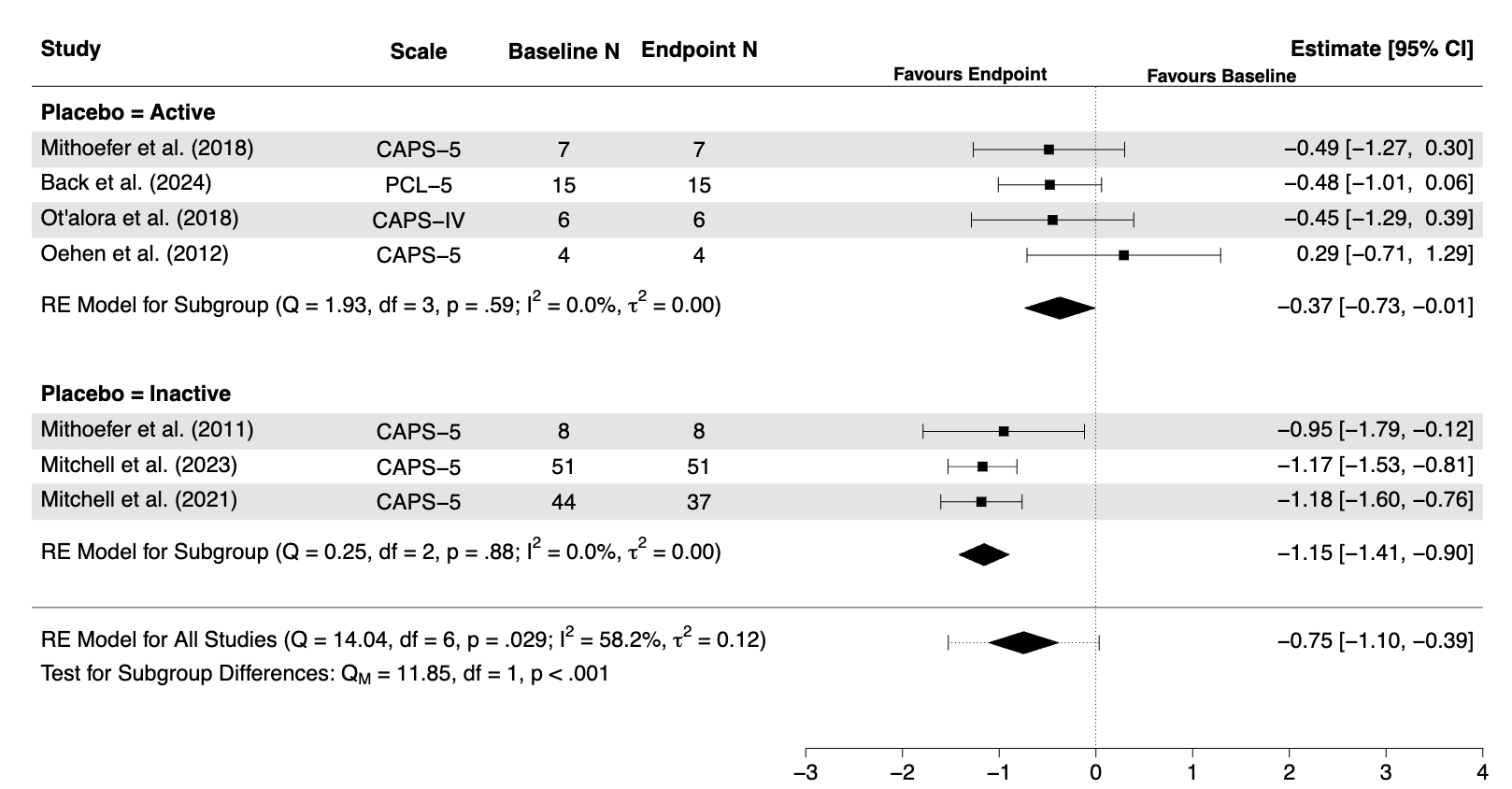


**Figure 7cS.** Sensitivity analysis excluding studies with high or some concerns of bias: forest plot of SMCC in PTSD symptoms within treatment group (k = 9).


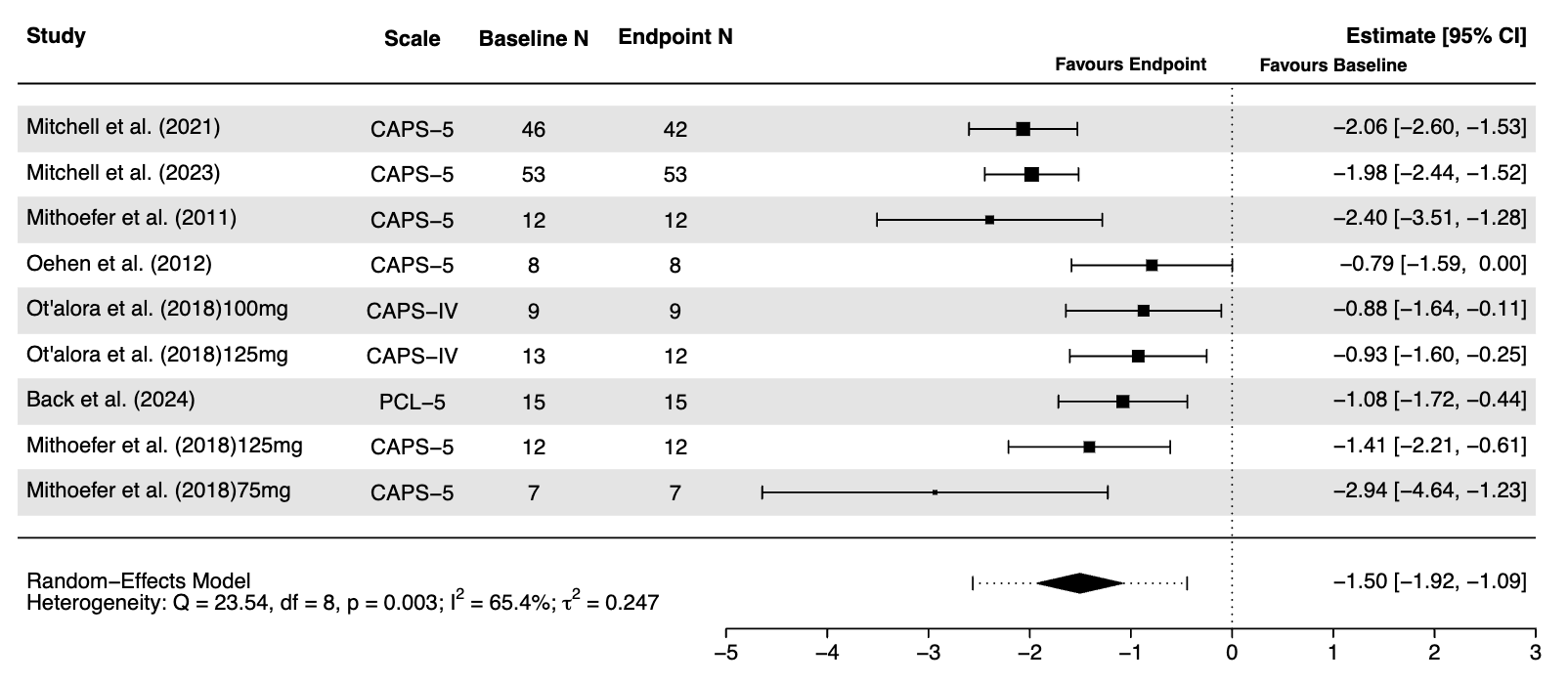


**Table 1S.** Risk of bias assessment of included studies.

| Author | Bias arising from the randomization process | Bias due to deviations from intended interventions | Bias due to missing outcome data | Bias in measurement of the outcome | Bias in selection of the reported result | Overall |
| --- | --- | --- | --- | --- | --- | --- |
| Back et al. (2024) | Low | Low | Low | Low | Low | Low |
| Bouso et al. (2008) | Low | High | Low | Some concerns | Some concerns | High |
| Goodwin et al. (2022) | Low | Low | Low | Low | Low | Low |
| Holze et al. (2023) | Low | Some concerns | Low | Low | Low | Some concerns |
| Mitchell et al. (2021) | Low | Low | Low | Low | Low | Low |
| Mitchell et al. (2023) | Low | Low | Low | Low | Low | Low |
| Mithoefer et al. (2011) | Low | Low | Low | Low | Low | Low |
| Mithoefer et al. (2018) | Low | Low | Low | Low | Low | Low |
| Oehen et al. (2013) | Low | Low | Low | Low | Low | Low |
| Ot’alora et al. (2018) | Low | Low | Low | Low | Low | Low |
| Palhano-Fontes et al. (2019) | Low | Low | Low | Low | Low | Low |
| Raison et al. (2023) | Low | Low | Low | Low | Low | Low |
| von Rotz et al. (2023) | Low | Low | Low | Some concerns | Low | Some concerns |
| Wolfson et al. (2020) | Low | Some concerns | Low | Low | Low | Some concerns |


**Table 2S.** Sensitivity analysis of direct between-arm meta-analytic results using various assumed within-study correlation (r) across outcomes.


| Outcomes (k) | r | SMD (95% CI) | I2 | 𝜏2 |
| --- | --- | --- | --- | --- |
| Depressive symptoms  (k = 13) | 0.3 | -0.74 [-1.04, -0.44] | 51% | 0.130 |
|  | 0.5 | -0.82 [-1.17, -0.47] | 60% | 0.223 |
|  | 0.7 | -0.96 [-1.42, -0.51] | 70% | 0.455 |
|  | 0.9 | -1.34 [-2.04, -0.64] | 82% | 1.334 |
| PTSD symptoms  (k = 10) | 0.3 | -0.85 [-1.10, -0.61] | 0% | 0 |
|  | 0.5 | -0.89 [-1.14, -0.65] | 0% | 0 |
|  | 0.7 | -0.95 [-1.20, -0.70] | 12% | 0 |
|  | 0.9 | -1.38 [-1.85, -0.92] | 54% | 0.230 |
| Anxiety symptoms (k = 5) | 0.3 | -0.61 [-0.89, -0.33] | 0% | 0 |
|  | 0.5 | -0.66 [ -0.94, -0.38] | 0% | 0 |
|  | 0.7 | -0.75 [-1.03, -0.47] | 10% | 0 |
|  | 0.9 | -1.27 [-1.87, -0.67] | 66% | 0.264 |

k: number of study arms; r: within-study correlation coefficient; SMD: standardized mean difference using Cohen's d; CI: confidence interval; I^2^: between-study heterogeneity; 𝜏^2^: between-study variance.

Bolded estimates indicate the primary analysis reported in the main manuscript.

**Table 3aS.** Sensitivity analysis of within-group results in control groups using various assumed within-study correlation (r) across outcomes.


| Outcomes (k) | r | SMCC (95% CI) | I2 | 𝜏2 |
| --- | --- | --- | --- | --- |
| Depressive symptoms  (k = 9) | 0.3 | -0.55 [-0.73, -0.36] | 30% | 0.021 |
|  | 0.5 | -0.58 [-0.77, -0.39] | 34% | 0.026 |
|  | 0.7 | -0.63 [-0.85, -0.42] | 44% | 0.040 |
|  | 0.9 | -0.81 [-1.13, -0.49] | 71% | 0.134 |
| PTSD symptoms  (k = 7) | 0.3 | -0.71 [-1.08, -0.34] | 61% | 0.138 |
|  | 0.5 | -0.75 [-1.10, -0.39] | 58% | 0.125 |
|  | 0.7 | -0.81 [-1.15, -0.46] | 53% | 0.104 |
|  | 0.9 | -0.93 [-1.26, -0.59] | 46% | 0.084 |
| Anxiety symptoms (k = 4) | 0.3 | -0.47 [-0.68, -0.27] | 0% | 0 |
|  | 0.5 | -0.51 [-0.71, -0.30] | 0% | 0 |
|  | 0.7 | -0.57 [-0.78, -0.37] | 0% | 0 |
|  | 0.9 | -0.84 [-1.22, -0.47] | 53% | 0.075 |

k: number of study arms; r: within-study correlation coefficient; SMCC: standardized mean change using change scores; CI: confidence interval; I^2^: between-study heterogeneity; 𝜏^2^: between-study variance.

Bolded estimates indicate the primary analysis reported in the main manuscript.

**Table 3bS.** Sensitivity analysis of within-group results in treatment groups using various assumed within-study correlation (r) across outcomes.

| Outcomes (k) | r | SMCC (95% CI) | I2 | 𝜏2 |
| --- | --- | --- | --- | --- |
| Depressive symptoms  (k = 13) | 0.3 | -1.18 [-1.52, -0.85] | 80% | 0.259 |
|  | 0.5 | -1.30 [-1.69, -0.92] | 84% | 0.362 |
|  | 0.7 | -1.53 [-2.03, -1.04] | 90% | 0.641 |
|  | 0.9 | -2.19 [-3.04, -1.34] | 96% | 2.080 |
| PTSD symptoms  (k = 10) | 0.3 | -1.36 [-1.77, -0.95] | 68% | 0.278 |
|  | 0.5 | -1.47[-1.86, -1.08] | 62% | 0.225 |
|  | 0.7 | -1.64 [-1.98, -1.30] | 43% | 0.118 |
|  | 0.9 | -2.00 [-2.26, -1.73] | 0% | 0 |
| Anxiety symptoms (k = 5) | 0.3 | -1.08 [-1.31, -0.84] | 0% | 0 |
|  | 0.5 | -1.16 [-1.40, -0.92] | 0% | 0 |
|  | 0.7 | -1.44 [-1.91, -0.97] | 58% | 0.149 |
|  | 0.9 | -2.15 [-3.17, -1.12] | 87% | 1.680 |

k: number of study arms; r: within-study correlation coefficient; SMCC: standardized mean change using change scores; CI: confidence interval; I^2^: between-study heterogeneity; 𝜏^2^: between-study variance.

Bolded estimates indicate the primary analysis reported in the main manuscript.
